# The impact of local control on overall survival after stereotactic body radiotherapy for liver and lung metastases from colorectal cancer: a combined analysis of 388 patients with 500 metastases

**DOI:** 10.1186/s12885-019-5362-5

**Published:** 2019-02-26

**Authors:** Rainer J. Klement, N. Abbasi-Senger, S. Adebahr, H. Alheid, M. Allgaeuer, G. Becker, O. Blanck, J. Boda-Heggemann, T. Brunner, M. Duma, M. J. Eble, I. Ernst, S. Gerum, D. Habermehl, P. Hass, C. Henkenberens, G. Hildebrandt, D. Imhoff, H. Kahl, N. D. Klass, R. Krempien, V. Lewitzki, F. Lohaus, C. Ostheimer, A. Papachristofilou, C. Petersen, J. Rieber, T. Schneider, E. Schrade, R. Semrau, S. Wachter, A. Wittig, M. Guckenberger, N. Andratschke

**Affiliations:** 1 Department of Radiation Oncology, Leopoldina Hospital Schweinfurt, Schweinfurt, Germany; 2 Department of Radiation Oncology, University Hospital Jena, Jena, Germany; 3 Department of Radiation Oncology, University Hospital Freiburg, Freiburg, Germany; 4 Strahlentherapie Bautzen, Bautzen, Germany; 5 Department of Radiation Oncology, Hospital Barmherzige Brueder, Regensburg, Germany; 6 RadioChirurgicum CyberKnife Suedwest, Goeppingen, Germany; 7 Department of Radiation Oncology Universitaetsklinikum Schleswig-Holstein, Luebeck, Germany; 8 Department of Radiation Oncology, University Medical Center Mannheim, University of Heidelberg, Mannheim, Germany; 9 Department of Radiation Oncology, Klinikum rechts der Isar- Technische Universitaet Muenchen, Munich, Germany; 10 Department of Radiation Oncology, University Hospital Aachen, Aachen, Germany; 11 Department of Radiation Oncology, University Hospital Muenster, Muenster, Germany; 12 Department of Radiation Oncology, Ludwig Maximilians University Munich, Munich, Germany; 13 Department of Radiation Oncology, University Hospital Heidelberg, Heidelberg, Germany; 14 Department of Radiation Oncology, University Hospital Magdeburg, Magdeburg, Germany; 15 Department of Radiotherapy and Special Oncology, Medical School Hannover, Hanover, Germany; 16 Department of Radiation Oncology, University of Rostock, Rostock, Germany; 17 Department of Radiation Oncology, University Hospital Frankfurt, Frankfurt, Germany; 18 Department of Radiation Oncology, Hospital Augsburg, Augsburg, Germany; 19 Department of Radiation Oncology, University Hospital Bern, Bern, Switzerland; 20 Department of Radiation Oncology, Helios Klinikum Berlin Buch, Berlin, Germany; 21 Department of Radiation Oncology, University Hospital Wuerzburg, Wuerzburg, Germany; 22 Department of Radiation Oncology, Faculty of Medicine and University Hospital Carl Gustav Carus, Technische Universität Dresden, Dresden, Germany; 23 Department of Radiation Oncology, University Hospital Halle, Halle, Germany; 24 Department of Radiation Oncology, University Hospital Hamburg, Hamburg, Germany; 25 Department of Radiation Oncology, University Hospital Basel, Basel, Switzerland; 26 Strahlenzentrum Hamburg, Hamburg, Germany; 27 Department of Radiation Oncology, Hospital Heidenheim, Heidenheim, Germany; 28 Department of Radiation Oncology, University Hospital of Cologne, Cologne, Germany; 29 Department of Radiation Oncology, Klinikum Passau, Passau, Germany; 30 Department of Radiotherapy and Radiation Oncology, Philipps-University Marburg, University Hospital Giessen and Marburg, Marburg, Germany; 31 Department of Radiation Oncology, University Hospital Zurich, University of Zurich, Rämistrasse 100, 8091 Zurich, Switzerland

**Keywords:** Colorectal cancer, Illness-death model, Liver metastases, Lung metastases, Tumor control probability, Stereotactic body radiation therapy

## Abstract

**Background:**

The aim of this analysis was to model the effect of local control (LC) on overall survival (OS) in patients treated with stereotactic body radiotherapy (SBRT) for liver or lung metastases from colorectal cancer.

**Methods:**

The analysis is based on pooled data from two retrospective SBRT databases for pulmonary and hepatic metastases from 27 centers from Germany and Switzerland. Only patients with metastases from colorectal cancer were considered to avoid histology as a confounding factor. An illness-death model was employed to model the relationship between LC and OS.

**Results:**

Three hundred eighty-eight patients with 500 metastatic lesions (lung *n* = 209, liver *n* = 291) were included and analyzed. Median follow-up time for local recurrence assessment was 12.1 months. Ninety-nine patients with 112 lesions experienced local failure. Seventy-one of these patients died after local failure. Median survival time was 27.9 months in all patients and 25.4 months versus 30.6 months in patients with and without local failure after SBRT. The baseline risk of death after local failure exceeds the baseline risk of death without local failure at 10 months indicating better survival with LC.

**Conclusion:**

In CRC patients with lung or liver metastases, our findings suggest improved long-term OS by achieving metastatic disease control using SBRT in patients with a projected OS estimate of > 12 months.

## Background

Colorectal cancer (CRC) is the third most common malignancy with a global burden expected to increase to 2.2 million new cases and 1.1 million deaths by 2030 [1]. Liver is the most common site of CRC metastasis and patients with liver metastases have been found to have a particularly poor prognosis with significantly reduced overall survival (OS) [2]. Still, in selected CRC patients with limited liver or lung metastases longer-term survival can be achieved with complete surgical resection of all metastatic lesions reaching 5-year OS rates up to 40% [3, 4]. Due to technological innovations and based on positive experiences with treating primary non-small cell lung cancer (NSCLC) patients, stereotactic body radiotherapy (SBRT) is increasingly adopted to treat pulmonary and hepatic metastases. So far, however, dose prescriptions have been mostly based on the experiences made with primary NSCLC in case of pulmonary metastases or on maximally tolerable doses for organs at risk in case of liver irradiation. Prospective studies investigating optimal dosing schedules are lacking for SBRT of extra-cranial metastases in general. For CRC metastases in particular, some have even claimed that the site of metastatic growth has an influence on radiosensitivity and thus local control (LC) probability [5]. To our knowledge, however, this has not yet been studied using sophisticated models of patient outcomes since no sufficient data has been collected so far.

An even more pressing question in this context is what value optimizing the probability of LC would have for longer-term OS. The rationale for using SBRT to treat extra-cranial metastases was partly based on the observation that patients with a limited number of pulmonary, hepatic or brain metastasis experienced survival benefits after complete surgical resection [6]. Hints for similar benefits of extra-cranial SBRT have so far been obtained in single-institutional studies of a small number of subjects [7]. Recently, a multicenter randomized phase II study for oligometastasized NSCLC could demonstrate a significantly improved progression-free survival with local treatment in patients responding to first-line chemotherapy versus maintenance chemotherapy alone [8]. The recently presented outcome data of the EORTC-NCRI CCSG-ALM Intergroup 40,004 trial could demonstrate for the first time a positive effect of a local ablative therapy in the form of radiofrequency ablation on OS in patients with liver metastases from CRC in addition to chemotherapy [9].

Sophisticated modeling of the putative importance of achieving LC for OS has not yet been performed, but may - with the scarce prospective data available - aid in the decision making on local treatment in oligometastasized patients. To address this highly relevant issue we have compiled the largest sample of colorectal lung and liver metastases treated with SBRT from two separate databases of the SBRT working group of the German Society for Radiation Oncology (DEGRO). This will be used to model the outcome after SBRT with regard to LC as well as OS with an emphasis on the role that the site of metastasis might play.

## Methods

### Data preparation

The analysis is based on pooled data from two large retrospective databases of SBRT treatments for pulmonary and hepatic metastases compiled from a total of 27 German and Swiss hospitals, all members of the DEGRO SBRT working group. All treatments performed on colorectal cancer patients were pooled into a new database, totaling 538 individual metastases treated with SBRT. Detailed descriptions of the separate databases have already been published [10, 11]. The multicenter data collection and analysis was approved by the respective local Ethics committees of the principle investigator’s institutions. For the current study, we excluded 38 metastases with no post-treatment evaluation of LC or OS, resulting in 500 metastases belonging to 388 patients. All clinical and treatment related variables relevant for the current analysis are compiled in Table 1.

**Table 1:** Variables and outcomes in our sample of 500 CRC metastases

Covariates and outcomes	n	Value	Liver	Lung	*p*-value
Sex	388				
*Male*	271	69.8	72.2	65.4	0.200
*Female*	117	30.2	27.8	34.6	
Age [years]	388	66 (24–93)	66 (24–93)	70 (38–36)	0.00399
Baseline Karnofsky index	283				
< 90	97	34.2	30.6	40.9	0.125
≥90	186	65.7	69.4	59.1	
Solitary metastasis	304				
Yes	110	36.2	33.2	40.7	0.184
No	194	63.8	66.8	59.3	
Number of treated metastases	388				
1	321	82.7	89.4	69.9	
2	42	10.8	8.2	15.8	
3	13	3.4	1.6	6.8	
4	7	1.8	0.4	4.5	
5	2	0.5	0.4	0.7	
6	3	0.8	0	2.3	
Tumor site	500				
*Liver*	291	58.2			
*Lung*	209	41.8			
Tumor volume [ccm] (gross tumor volume, GTV)	342	9.20 (0.07–699)	26.0 (0.8–699)	3.1 (0.07–268)	< 0.0001
Chemotherapy prior to SBRT	430				
*Yes*	332	77.2	84.5	68.5	< 0.0001
*No*	98	22.8	15.5	31.5	
Dose calculation algorithm	496				
*Pencil beam*	199	40.1	56.4	17.7	< 0.0001
*Advanced*	297	59.9	43.6	82.3	
Motion management	500				
*Free breathing*	347	69.4	75.3	73.7	0.755
*Advanced*	153	30.6	24.7	26.3	
BED_iso_ [Gy_10_]	500	126.9 (37.5–309.4)	124.8 (37.5–234.5)	141.1 (39.4–309.4)	< 0.0001
Outcomes	388				
*Local failure and censored prior to death*	28	7.2	7.5	6.8	< 0.0001
*Local failure and death*	71	18.3	24.3	6.8	
*Death without local failure*	133	34.3	36.5	30.0	
*Censored prior to local failure or death*	156	40.2	31.8	56.4	

Values for continuous variables are given as median (range), those for categorical variables as frequencies in percent. The p-values refer to testing for differences between liver and lung metastases with respect to the variable specified in each row. The Wilcoxon rank sum test and Fisher’s exact test were used to compare continuous and categorical variables, respectively. The variable “solitary metastasis” was coded as “yes” if only lung or liver was involved by a singular metastasis. Beyond this information, the exact location and number of additional metastases has not been encoded

Local recurrence for the SBRT treated lesions was defined as either reappearance after complete remission or re-growth after initial partial response to SBRT in follow-up CT or MRI scans. PET-CT scans were used by some centers in equivocal cases to confirm local recurrence. In case that death and local failure were recorded at the same date (this was the case for only 2 metastases), we adopted the convention that death happened first and treated LC as censored [12]. For modeling, all prescriptions were converted to biologically effective doses at the isocenter defined as ${\mathrm{BED}}_{\mathrm{iso}}= nd\left(1+\frac{d}{\alpha /\beta}\right)$ where *n* is the number of fractions, *d* the fraction dose at the isocenter and *α*/*β* assumed as 10 Gy.

The binary variable “chemotherapy prior to treatment” was set to 1 if a patient had received chemotherapy prior to SBRT at least once, and maximum tumor volume did correspond to the largest metastasis treated. Table 1 provides details on all covariates and outcomes considered for modeling.

Two hundred fifty-five patients were treated for a total of 291 liver and 133 patients for a total of 209 lung metastases. In the dichotomization of the dose calculation algorithm, “Advanced” refers to either Collapsed Cone, Analytical Anisotropic Algorithm or Monte Carlo based algorithms.

### Model description

**Fig. 1:**
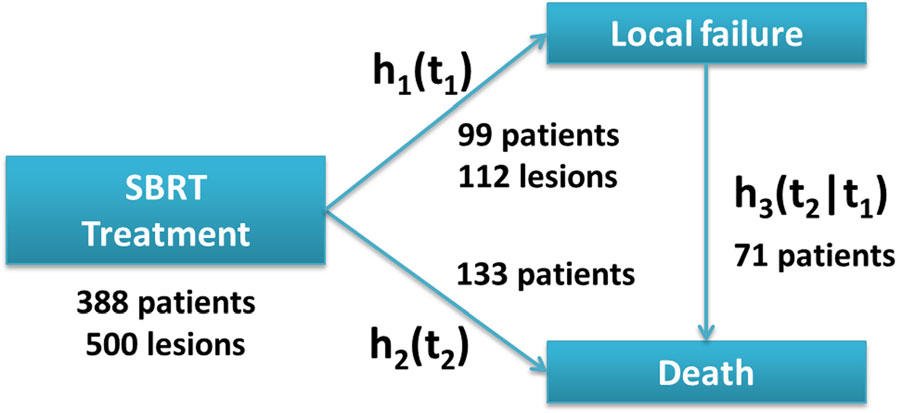
Conception of the illness-death modeling framework applied to the study of local failure and death in metastatic rectal cancer patients treated with SBRT. Starting from the state “SBRT treatment”, patients can either transition into the state “Local failure” (the non-terminal event occuring at time *T*_1_) or “Death” (the terminal event occurring at time *T*_2_). A third transition from “Local failure” to “Death” is also possible, but not vice versa. The rates at which patients transition from one state to the other are specified by three corresponding hazard functions that we model using Eqs. (1–3). *h*_1_(*t*_1_) is the hazard rate for local failure from SBRT at a given point in time *t*_1_, given that neither local failure or death have occurred before *t*_1_. *h*_2_(*t*_2_) is the hazard rate for death after SBRT at a given point in time *t*_2_, given that neither local failure nor death have occurred before *t*_2_. Finally, *h*_3_(*t*_2_ ∣ *t*_1_) is the hazard rate of death at a given time point *t*_2_ given that local failure has been observed at *T*_1_ = *t*_1_ and that death has not occurred before *t*_2_

Our main interest lies in the relationship between LC and OS. We model these two outcomes within the context of a multistate model, or more specific, a so-called illness-death model [13] where death can occur after local failure but not vice versa. In the illness-death model there are three possible transitions (Fig. 1): (1) treatment to relapse; (2) treatment to death; (3) relapse to death.

We assumed proportional cause-specific hazards and included frailty terms to account for possible heterogeneity between hospitals. For transition (1) the unit of interest is the treated lesion. Clustering of metastases within patients was neglected due to the very small number of metastases associated with each patient. For transitions (2) and (3) the unit of interest was the patient. For all three transitions the clustering of patients within hospitals was accounted for using a shared frailty model [14]. In case of multiple treatments we define the first treatment as the time origin and time of the first relapse as the failure time.

Let *T*_*ijk*1_ denote the time from treatment until relapse for metastasis *k* belonging to patient *j* from hospital *i* with *k* = 1, … , *n*_*ij*_, *j* = 1, … , *s*_*i*_ and *i* = 1, … , *h*. Furthermore, let *T*_*ij*2_ denote the time from first treatment until death for patient *j*. The three possible transitions are then specified by the three cause-specific hazard functions:

1 $$ {h}_1\left({t}_{ijk1}|{w}_{i1},{\boldsymbol{X}}_{ijk1}\right)={h}_{01}\left({t}_{ijk1}\right)\ \exp \left({\boldsymbol{X}}_{ijk1}^T{\boldsymbol{\beta}}_1+{w}_{i1}\right),\kern6.50em {t}_{ijk1}>0 $$

2 $$ {h}_2\left({t}_{ij2}|{w}_{i2},{\boldsymbol{X}}_{ij2}\right)={h}_{02}\left({t}_{ij2}\right)\ \exp \left({\boldsymbol{X}}_{ij2}^T{\boldsymbol{\beta}}_2+{w}_{i2}\right),\kern9em {t}_{ij2}>0 $$

3 $$ {h}_3\left({t}_{ij2}|\left\{{t}_{ij k1}\right\},{w}_{i3},{\boldsymbol{X}}_{ij3}\right)={h}_{03}\left({t}_{ij2}|\left\{{t}_{ij k1}\right\}\right)\exp \left({\boldsymbol{X}}_{ij3}^T{\boldsymbol{\beta}}_3+{w}_{i3}\right),\kern0.5em {t}_{ij2}>{t}_{ij k1} $$

Here ***w***_*i*_ = (*w*_*i*1_, *w*_*i*2_, *w*_*i*3_) is a vector of hospital-specific random effects (each belonging to one of the three transitions) and ***β***_*g*_, *g* ∈ {1, 2, 3} denotes the transition-specific fixed effects. The random effects ***w***_*i*_ were assumed to stem from a Gaussian distribution with mean 0 and variance ${\sigma}_i^2$.

The hazard *h*_3_ in Eq. (3) defines the rate of death of patient *j* from hospital *i* following the occurrence of local failure at time *T*_*ijk*1_ = *t*_*ijk*1_. This hazard principally depends on the set of failure times ${\left\{{t}_{ij k1}\right\}}_{k=1,\dots, {n}_{ij}}$ of the *n*_*ij*_ metastases of patient *j*. To simplify we apply the Markov assumption, *h*_3_(*t*_*ij*2_| {*t*_*ijk*1_}) = *h*_3_(*t*_*ij*2_), in which the hazard of death does not depend on the particular time of tumor recurrence. This can be interpreted as stating that a patient’s risk of death at any time after the first SBRT is initially described by the hazard in Eq. (2), but if and when a local relapse has occurred takes on the form of Eq. (3) [13]. The hazard *h*_3_, which is estimated using patients that have experienced local relapse, is theoretically defined for every patient beginning from the earliest time point that a local relapse has been recorded in our data (which was 2 months). The Markov assumption is useful for inferring the general importance of achieving LC for a patient’s probability of OS. In this case, the hazards *h*_2_(*t*_*ij*2_) and *h*_3_(*t*_*ij*2_) correspond to the hazards of death at time *t*_*ij*2_ given that death has not occurred before *t*_*ij*2_ and all metastases have been controlled or not before *t*_*ij*2_, respectively. The so-called explanatory hazard ratio *h*_3_/*h*_2_ characterizes the dependence between *T*_*ijk*1_and *T*_*ij*2_. If *h*_3_/*h*_2_ = 1, the occurrence of *T*_*ijk*1_ has no effect on the hazard of dying at *T*_*ij*2_, while when *h*_3_/*h*_2_ > 1 at *t*_*ij*2_ this indicates that for a fixed value of the frailty and covariates, the risk of death is higher if a relapse had occurred before *t*_*ij*2_ [15].

Alternatively, one could think of treating local failure as a time-dependent covariate *Z*_*j*_(*t*) which takes on value 0 as long as LC is achieved, but jumps to 1 if local failure occurs in patient *j*. In this case, the hazard of death would be $h\left({t}_{ij}|{w}_i,{\boldsymbol{X}}_{ij}\right)={h}_0\left({t}_{ij}\right)\ \exp \left({Z}_j(t){\beta}_0+{\boldsymbol{X}}_{ij}^T\beta +{w}_i\right)$ so that *h*_2_ and *h*_3_ would have the same baseline hazard, their ratio would be given as *h*_3_/*h*_2_ = exp(*β*_0_), and *h*_1_ would be left unspecified. These restrictions do not apply to the illness-death model whose structure naturally accounts for the relation between LC and OS.

Models were fit using maximum integrated partial likelihood estimation by the Laplace approximation with the R package coxme. The refine.n option was used to confirm the goodness of the Laplace approximation via Monte Carlo control sampling.

Given a specification of the baseline hazard function, the probability of local failure or death between time *t* and horizon time *t* + *w* can be estimated. We therefore complemented the analysis by approximating the baseline hazard function on the basis of cubic M-splines that were fit using the frailtypack package [16]. A lognormal frailty was used which is equivalent to Gaussian random effects [14]. The frailtypack routine uses maximum penalized likelihood estimation based on the robust Marquardt algorithm [16]. For each transition, the number of knots for the splines was set to 9 and the optimal smoothing parameter κ in the penalized log likelihood was estimated by cross-validation [16].

### Variable selection and model preparation

For survival regression, it is generally recommended to restrict the number of covariates to approximately the number of events divided by 15 [17]. We tried to use this as a constraint to select an appropriate number of covariates for each of the three transitions (Eqs. 1–3) from the full set shown in Table 1 based on clinical interest and knowledge from previous modeling studies.

Chemotherapy prior to SBRT treatment (missing for 14% of metastases) and tumor volume (missing for 31.6%) were judged as putative prognostic factors for LC, and baseline Karnofsky performance score (KPS, missing for 27% of patients) as well as presence of multiple metastases (missing for 21.6%) as possible prognostic factors for OS. We imputation by chained equations (mice) [18] to impute the missing values for these covariates in order to maximize the number of cases for modeling. The ideal set of predictors for each variable was determined with help of the mice package function quickpred by requiring a minimal correlation of 0.2 and minimum proportion of usable cases of 0.25. For 99 of the 158 lesions with unknown tumor volume, the PTV volume was available and used in the imputation. Sensitivity to the particular imputation of the variables was checked by performing a total of 50 imputations, each time refitting the models and pooling the results together using the pool function from the mice package in R [18].

All covariates except BED_iso_ and tumor volume were categorized to 0/1; BED_iso_ and tumor volume were standardized by subtracting the mean and dividing by two standard deviations [19].

### Statistical tests

Fisher’s exact test and the Wilcoxon rank sum test were used to compare categorical and continuous variables, respectively, between liver and lung metastases. Two-sided Wald tests were used to obtain *p*-values for regression coefficients.

## Results

In total, 388 patients with 500 metastatic lesions (lung *n* = 209, liver *n* = 291) were included and analyzed. Most frequent dose prescriptions were 5 × 7 Gy @65% isodose line (11.6% of metastases), 1 × 24Gy @80% (7.4%) and 3 × 12.5 Gy @65% (5.4%). Chemotherapy was significantly more often administered prior to SBRT of liver (84.5%) compared to lung (68.5%) metastases (Table 1). Also, lung metastases received significantly higher BED_iso_ than liver metastases. Tumor volumes were significantly larger in liver compared to lung metastases, and advanced dose calculation algorithms were used significantly more often for planning treatment of the latter. Due to these differences the site of tumor location was included as an important confounding factor into each of the following models.

Median follow-up time for LC was 12.1 months (range 0.03–95.7) and 17.8 months (0.16–151.8) for OS. There were 3 metastases in 2 patients that were lost to LC assessment directly after the end of SBRT treatment which explains the short FU duration of 0.03 months; both patients died 21 and 53 days after beginning of SBRT, respectively. In total, 99 patients with 112 lesions experienced local failure. Seventy-one of these patients have died after experiencing local failure. Median survival time was 27.9 months (95% CI 24.4–31.8) in all patients and 25.4 months (95% CI 23.6–33.0) versus 30.6 months (95% CI 24.5–37.4) in patients with and without local failure after SBRT (*p* = 0.19), respectively. Local relapses were recorded in 31.3% of liver metastases compared to 10.0% of lung metastases (*p* < 0.0001).

Table 2 provides an overview of all covariates selected for modeling each transition together with their regression coefficients. In the multivariable analyses, smaller tumor volumes, advanced motion management and dose calculation techniques were non-significantly associated with higher LC rates; significantly better tumor control was found for lung metastasis compared to liver metastases, no prior chemotherapy and higher BED_iso_.

**Table 2:** Covariates selected for modeling each transition (Eqs. 1–3) and their estimated regression coefficients expressed as hazard ratios

Transition	Treatment to local failure (1)			Treatment to death (2)			Local failure to Death (3)		
Covariates	exp(β)	95% CI	*p*-value	exp(β)	95% CI	*p*-value	exp(β)	95% CI	*p*-value
Sex: Female				0.97	0.65–1.44	0.867			
Age ≥ 66				1.13	0.75–1.69	0.564			
KPS ≥ 90				0.47	0.29–0.78	0.0037	1.30	0.56–3.02	0.534
Tumor site: Lung	0.42	0.25–0.70	0.0010	0.89	0.56–1.41	0.611	1.30	0.56–3.05	0.537
Solitary metastasis: Yes				0.83	0.53–1.29	0.405	0.55	0.23–1.30	0.174
Number of treated metastases > 1				0.96	0.59–1.56	0.861			
Chemotherapy prior to SBRT: Yes	3.64	1.58–8.36	0.0024	1.19	0.71–1.98	0.508	0.19	0.04–0.84	0.028
Tumor volume	1.20	0.89–1.63	0.232	1.99	1.47–2.69	< 0.0001	2.12	1.25–3.58	0.0053
Motion management: Advanced	0.81	0.49–1.34	0.411						
Dose calculation: Advanced	0.86	0.55–1.34	0.497						
BED_iso_	0.39	0.25–0.64	0.00013						

If a variable was not used as a covariate for modeling the hazard of a particular transition, its corresponding cell has been left empty. For Transition (2), tumor volume refers to the maximum tumor volume of all treated metastases within a particular patient

*KPS* Baseline Karnofsky performance status

**Fig. 2:**
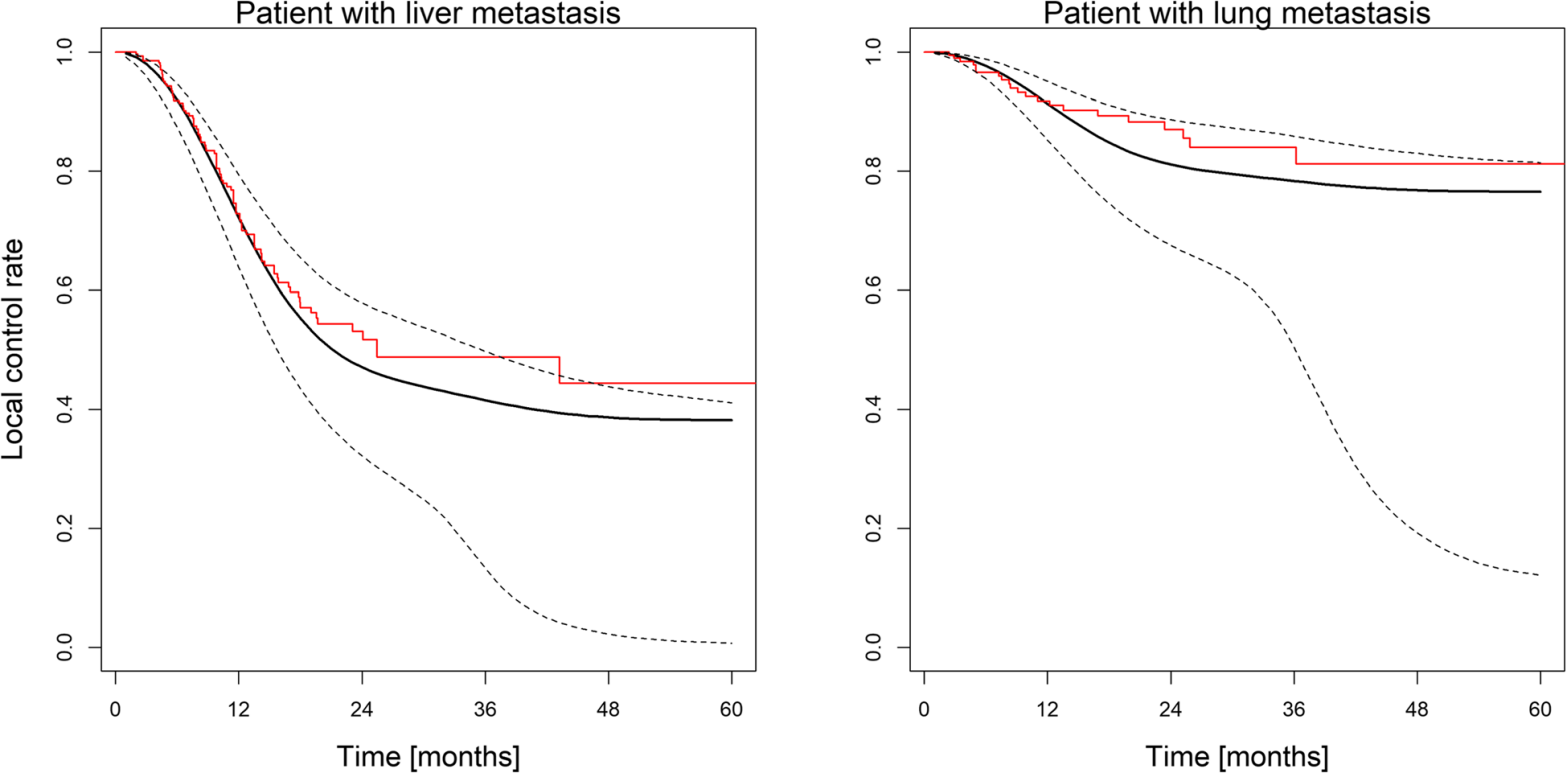
Tumor control probability predictions for treatment of a lung and liver metastasis with an average dose of BED = 132 Gy_10_. The left panel shows the prediction for a liver metastasis, the right panel for a lung metastasis. The black dotted line is a 95% CI for the black solid line based on 500 Monte Carlo samples. In both cases the other treatment characteristics (motion management, dose calculation algorithm, chemotherapy prior to SBRT) are the same. The Kaplan-Meier tumor control probability curves for liver and lung metastases are shown in red for comparison

Figure 2 shows the Kaplan-Meier tumor control probability (TCP) curves for both liver and lung metastases together with the predictions of the lognormal frailty model for an average treatment. In this context, “average” refers to the mean and most frequent value for continuous and categorical variables, respectively, of lung and liver metastases. The model predicts that for a 90% TCP at 2 years, BEDiso of 99 Gy_10_ (lung) and 187 Gy_10_ (liver) would be sufficient with no prior chemotherapy, but 211 Gy_10_ (lung) and 300 Gy_10_ (liver) would be needed if prior chemotherapy would have been given (Table 3).

**Table 3:** BED_iso_ converted to clinically applicable dose fractionation schedules to achieve at least 90% local control at 2 years of CRC metastases

Tumor location	No prior Chemo		Prior Chemo	
Lung	99 ± 15 Gy_10_ BED_iso_	3 × 9 Gy @ 65%	211 ± 19 Gy_10_ BED_iso_	3 × 15 Gy @ 65%
		8 × 5 Gy @ 65%		5 × 10.5 Gy @ 65%
Liver	187 ± 19 Gy_10_ BED_iso_	3 × 14 Gy @ 65%	300 ± 39 Gy_10_ BED_iso_	3 × 18 Gy @ 65%
		5 × 10 Gy @ 65%		5 × 13 Gy @ 65%

For patients without local recurrence, baseline KPS and maximum tumor diameter were found to be significant for OS. If and when local failure occurred, maximum tumor diameter remained highly associated with worse OS, while there was a weaker, yet significant association for chemotherapy prior to SBRT to improve OS (Table 2). We found that because of the significantly larger sizes of liver metastases, controlling for maximum tumor diameter was important for diminishing an otherwise significant effect of liver metastases predicting worse OS in transition 2.

**Fig. 3:**
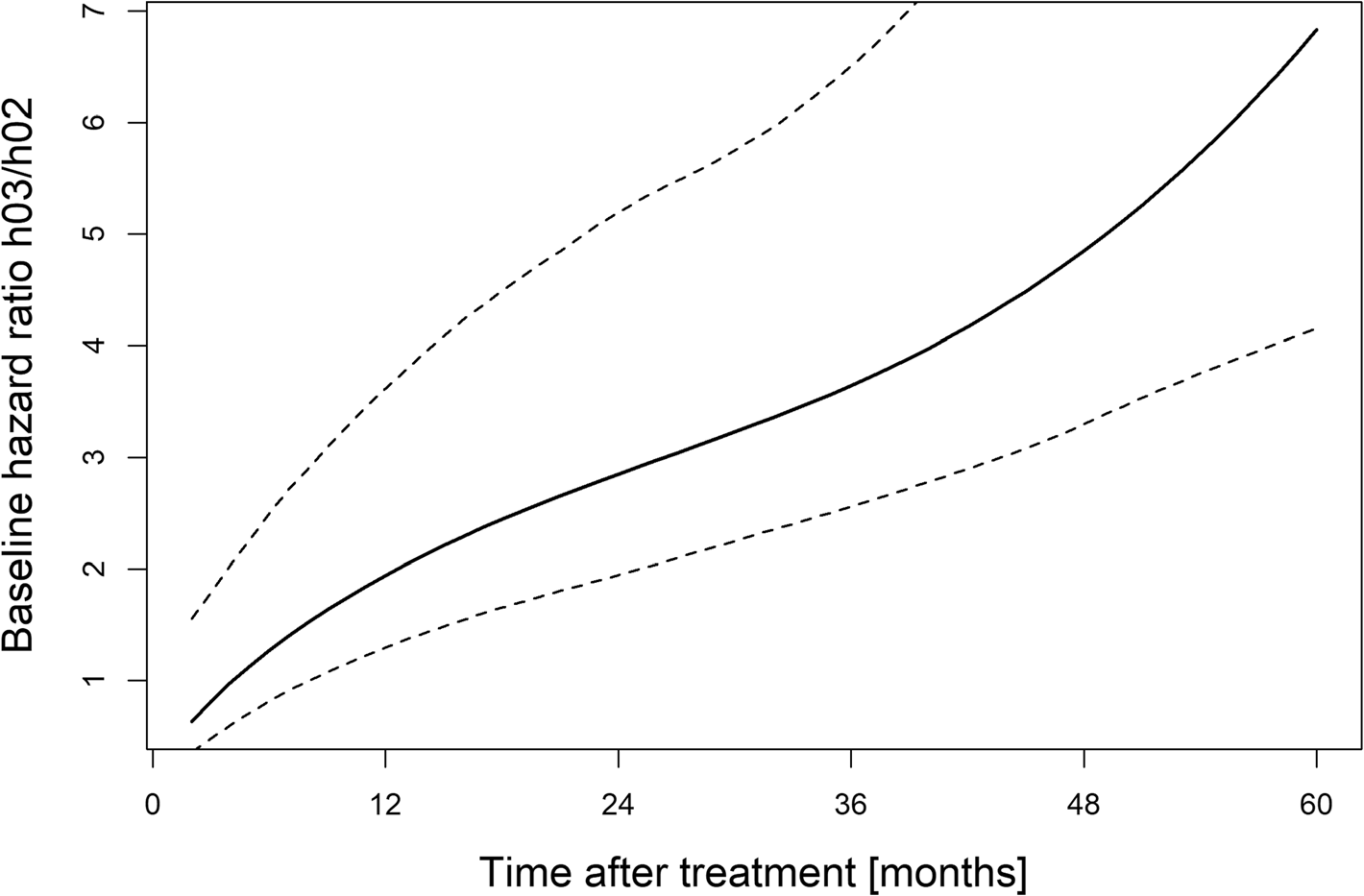
Baseline hazard ratio between transitions 3 and 2 as a function of follow-up time after treatment. Ratios greater than 1 indicate a greater risk of death if a patient has experienced a local recurrence prior to the time considered. The dashed lines indicate the 95% confidence band based on 500 Monte Carlo simulations of the baseline hazards. A very similar trend is observed when computing the baseline hazard ratio for a lung metastasis patient (coded with tumor site = 1), although the confidence bands are wider (not shown)

Figure 3 shows the baseline hazard ratio (*h*_03_/*h*_02_) between death after local recurrence (transition 3) and death without local recurrence (transition 2) as a function of time starting at 2 months, the earliest time of recurrence recorded in our database. The baseline hazards correspond to the hazard of death when all fixed and random effects are zero. It can be clearly seen that from about 10 months on, the baseline risk of death after local failure significantly exceeds the baseline risk of death without local failure, and the ratio increases with time indicating better survival after LC is achieved. This implies that if the overall prognosis is judged to be beyond 1 year LC is a decisive factor for OS.

**Fig. 4:**
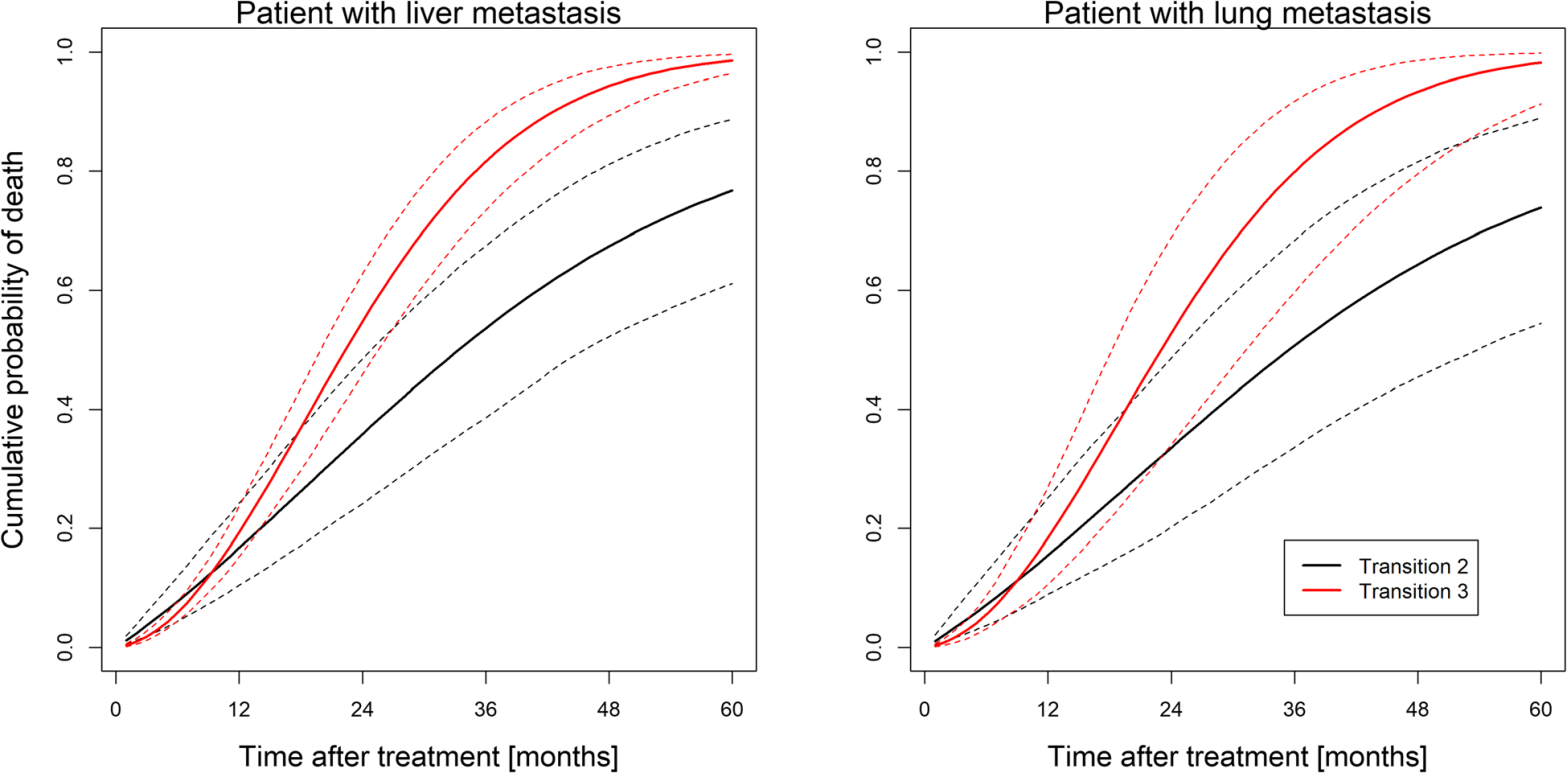
Cumulative probability of making transitions 2 (black) and 3 (red) as a function of follow-up time after treatment. Predictions are for an average patient (male, KPS ≥ 90, age < 66 years, one metastasis, given chemotherapy) with a liver (left panel) or lung (right panel) metastasis, respectively. 95% confidence bands based on 500 Monte Carlo samples are shown as dotted lines. All predictions are averaged over different imputations of the chemotherapy covariate. Note that after some short initial time the probability of transition 3 starts to exceed that of transition 2, indicating a higher probability of death if the metastasis has not been controlled

Figure 4 shows the predicted cumulative probability of dying without (transition 2) or after (transition 3) experiencing local recurrence. Two predictions for an average patient with either lung metastasis (right panel) or liver metastasis (left panel) are shown. The cumulative death probability for dying after local recurrence is almost equal for lung and liver metastases, with slightly differing 95% confidence bands indicating a similar effect of local recurrence on OS. In contrast, the cumulative probability of dying without experiencing local recurrence is higher in patients with liver than in patients with lung metastases indicating a generally worse prognosis of patients with liver compared to lung metastases. Still, in both cases the probability of making the transition 3 (death after local recurrence) quickly exceeds that for transition 2 (death without local recurrence), consistent with the behavior of the baseline hazard ratio shown in Fig. 3, underlining the impact of LC on OS.

## Discussion

Although Hellman and Weichselbaum coined the concept of oligometastases as a distinct state with its own biology in 1995 [20], it only recently started to be more thoroughly investigated in the context of SBRT [7, 21]. Nevertheless, up until recently, there was no clinical proof that oligometastatic patients really do benefit from local interventions such as surgery or SBRT. A possible hint for a benefit could be derived from surgical series indicating worse OS after microscopic incomplete resection compared to complete resection, indicating the need for LC of the respective metastases [22]. With the outcome of the EORTC-NCRI CCSG-ALM Intergroup 40,004 trial, the first prospective data showing a positive effect of a local ablative treatment on OS – in this case radiofrequency ablation - in patients with liver metastases from CRC became available [9].

On the other hand, SBRT series on oligometastatic patients included most frequently a mixture of histologies rendering comparison with surgical series difficult (e.g.,[6, 21–26]). The intend of this analysis therefore was to focus on metastatic CRC patients only, to avoid bias by histology and to investigate the effect of LC on OS after SBRT for lung or liver metastases. In addition, we wanted to investigate the effect of metastatic site (liver, lung) on tumor control rates, as there have been observations that site-specific differences in the response of CRC metastases to ionizing radiation might exist [27–33].

Our analysis is the first to specifically address the question whether the site of colorectal cancer metastases plays a role in the response to SBRT treatment, but also and even more importantly whether LC also translates into improved OS. Applying an illness-death type multistate model to a total of 500 SBRT treatments of CRC metastases, we studied differences between lung and liver metastases with respect to LC and OS and the interplay between both outcomes. The structure of the illness-death model naturally accounts for the relation between LC and OS which is not the case if LC would be treated as a time-dependent covariate.

### Dose-response analysis and factors influencing local control

For achieving LC, we identified radiation dose or, more precisely, BED_iso_ as the most important variable. However, the dose-response relationship was strongly influenced by tumor site and whether chemotherapy had been given prior to SBRT. The results are also in line with our previous modeling studies of the full liver and lung metastases cohorts from which the data analyzed here had been extracted: Without taking any confounding factors into account, the BED needed to achieve 90% TCP after 1 year was estimated as 192 Gy_10_ (SE 37 Gy_10_) for liver metastases [11], but 110 Gy_10_ (SE 17 Gy_10_) for lung metastases [34]. Further data providing evidence for an influence of tumor site on LC have been published by Ahmed et al. [5] who reported 100% LC rates at 2-years after SBRT treatment of CRC lung metastases, but only 73.0% in liver metastases of similar sizes using the same prescription of 60 Gy in 5 fractions. Although their number of 29 lesions in total was small, these differences were statistically significant (*p* = 0.026) and attributed to differences in the parenchyma and vasculature of liver and lung tissue [5]. A significantly larger *α*/*β* ratio for hepatic compared to lung metastases, as recently estimated in a meta-regression study by Klement [35], would be consistent with such an explanation. In contrast to the assumption of different site dependent radiation sensitivity by Ahmed et al., another interpretation for the difference in LC between lung and liver metastases could be the challenges in target volume delineation, image guided radiation delivery and motion management technique. While this is supported by a recent dose-response analysis of our large SBRT database of lung and liver metastases, indicating that the motion management technique significantly impacts LC for liver, but not lung metastases ([11] and unpublished data), the significance of tumor site persisted even after controlling for motion management in our analysis. One limitation of this analysis is that information on the within-organ location of individual metastases was lacking; in the full lung metastases data set peripheral tumor location (compared to central location) was associated with improved TCP [34].

Our finding of chemotherapy prior to SBRT being a negative predictive factor for LC is certainly intriguing and needs further investigation. However, we believe that this finding may explain the frequent notion that CRC metastases (which are frequently heavily pre-treated) are more radioresistant compared to other histologies [11]. Unfortunately, detailed information on the type of chemotherapy was lacking, but in particular adjuvant oxaliplatin (FOLFOX) therapy has been shown to be associated with worse outcomes in patients who later developed liver metastases, consistent with the increased accumulation of new somatic mutations and acquirement of therapy resistance [36]. Another explanation might be that patients having received chemotherapy prior to SBRT would be more likely to have node-positive primaries, indicative of more aggressive tumors.

### The impact of local control on overall survival

The dependence of OS on LC is the most relevant finding of this study. For the first time reported in SBRT treated metastatic patients, a survival benefit was observed, if LC of individual metastases has been achieved. Our results are consistent with surgery outcomes showing significantly longer 5-year OS after R0 compared to R1 resection of hepatic metastases in CRC patients with or without prior chemotherapy [22].

As Figs. 3 and 4 show, the probability of dying after a local relapse soon exceeds that of dying if no relapse has occurred. However, this difference becomes apparent only after about 10 months of follow-up (separation of the transition curves in Fig. 4), implying that LC impacts OS if the projected prognosis is beyond this time point. Conversely, if the projected survival is less than 10 months, achieving LC is clinically not relevant.

This finding directly relates to two important aspects to achieve a possible survival benefit: patient and radiation dose selection.

Selection of an appropriate radiation dose is directly related to a previous analysis by our group developing a model of TCP depending on both dose and time [34]. In this model, the dose needed for a certain probability of LC was time dependent: the longer the time interval the higher the respective BED necessary for the same TCP. Translated to our current findings this implies that if the projected prognosis is beyond 12 months, BED needs to be high enough in order to assure long-term LC and improved OS. Therefore, beyond an organs-at-risk adapted dose selection, a time-adjusted BED with clinically relevant LC (e.g. 90% TCP at 2 years) should be employed in metastasized CRC patients with presumed longer-term survival. Examples of such dose prescriptions based on our model are given in Table 3. The high doses predicted by our model as necessary to achieve high long-term TCP in chemotherapy pre-treated metastases may be very difficult to achievable clinically due to normal tissue dose constraints, especially in the liver. However, besides the fact that these doses are not necessarily generalizable to other target populations, they primarily highlight the complicated interplay between patient prognosis and dose escalation.

**Fig. 5:**
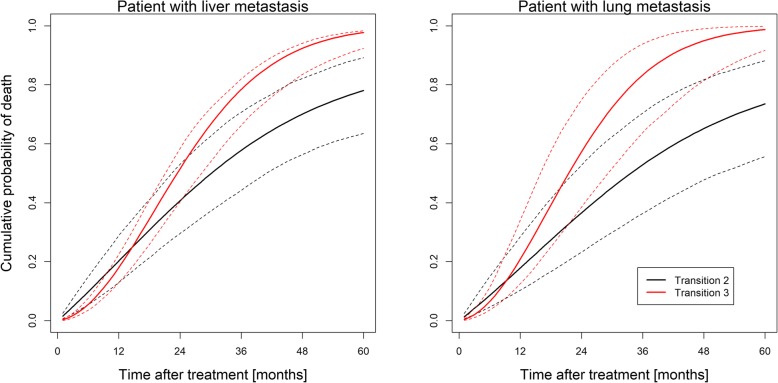
Same as Fig. 4, but based on an analysis using only the subset of 311 metastases with no missing variables. Note that specifically for lung metastases patients, the confidence bands are somewhat narrower than for the imputed dataset which could be explained by the larger variation induced through pooling 50 different imputated datasates together as was done in Fig. 4

That the results of this retrospective study are specific to our cohort and not necessarily generalizable to a different target population poses a major limitation for the interpretation of our results. While similar analyses in different cohorts and future prospective trials need to be conducted, specifically the causal claim that LC influences OS would be further supported by studying potential underlying mechanisms [37]. Another limitation is that our cohort lacked detailed information on the pre-SBRT treatment history of individual patients, in particular concerning types of chemotherapy and local therapies such as previous liver or lung resections. It follows that patients probably had varying prognoses of both LC and OS at the time of SBRT depending on factors that we were not able to account for in our model. A prospective study with a more balanced patient cohort, possibly as a randomized trial applying different fractionation schedules, would be very helpful for confirming our finding that achieving long-term LC has a positive impact on OS. Finally, a large number of metastases had at least one missing variable (189 or 37.8% of the sample) which we decided to impute and which in principal could have influenced our results. However, re-analyzing the sample of 311 metastases with complete entries, we obtained qualitatively similar results to the main analysis, in particular regarding the importance of predictors of transitions 1–3, and the impact of LC on OS (Fig. 5).

Current patient selection criteria for local ablative treatment are based on surgical analyses of oligometastatic CRC patients with liver and lung metastases and relate clinical parameters with prognosis. In optimally selected candidates OS rates of up to 40% at 5 years can be achieved for those patients. Conversely, even patients who only fulfill a subset of these criteria may benefit from local intervention. Therefore, a finer grained approach would be desirable to estimate an individual’s projected survival based on a combination of clinical parameters to guide the optimal decision regarding local ablative treatment. To this end, we recently developed a nomogram for oligometastatic lung disease treated with SBRT based on clinical parameters: KPS, type of the primary tumor, control of the primary tumor, maximum diameter of the largest treated metastasis and number of metastases (1 versus > 1) [38]. These parameters are readily available and the nomogram allows deriving 4 prognostic groups which could serve as guidance for patient selection regarding local ablative treatment of oligometastatic disease. Still, validation of this nomogram in other metastatic disease sites is warranted to generalize its applicability in oligometastatic disease.

## Conclusion

Utilizing a large patient cohort from a multi-institutional database and focusing on CRC patients with lung or liver metastases, the variables radiation dose, tumor site and pre-SBRT chemotherapy could be identified as predictive factors for LC after SBRT. Most importantly, application of an illness-death model revealed an association between achieving LC with SBRT and OS: patients without local recurrence had a significantly lower risk of death when adjusting for other influencing factors such as tumor site (lung vs. liver), maximum tumor volume or performance status. However, this was only observed in patients with a projected OS estimate of approximately 12 months or more, underlining the importance of proper patient selection for dose-intensified SBRT.

## Abbreviations

BED: Biologically effective dose
CI: Confidence interval
CRC: Colorectal cancer
DEGRO: German Society for Radiation Oncology
GTV: Gross tumor volume
KPS: Karnofsky performance score
LC: Local control
NSCLC: Non small cell lung cancer
OS: Overall survival
PTV: Planning target volume
SBRT: Stereotactic Body Radiation Therapy
SE: Standard error
TCP: Tumor control probability
